# Supplementary material for: BRCA1–BARD1 combines multiple chromatin recognition modules to bridge nascent nucleosomes
Source: Nucleic Acids Res. 2023 Oct 12;51(20):11080–103. doi: 10.1093/nar/gkad793 (PMC10639053; doi:10.1093/nar/gkad793)
Supplement: gkad793_Supplemental_Files [file gkad793_supplemental_files.zip › BRCA1-BARD1_nucleosome_interaction_Supplemental_figures_v15.pdf]

BRCA1-BARD1 combines multiple chromatin recognition modules to bridge nascent nucleosomes

SUPPLEMENTARY FIGURES

Hayden Burdett[#1], Martina Foglizzo[#*2], Laura J. Musgrove[2], Dhananjay Kumar[1], Gillian Clifford[1], Lisa J. Campbell[2], George R. Heath[3], Elton Zeqiraj[*2] and Marcus D. Wilson[*1]

[1]Wellcome Centre for Cell Biology, University of Edinburgh, Michael Swann Building, Kings Buildings, Mayfield Road, Edinburgh, EH9 3JR, UK
[2]Astbury Centre for Structural Molecular Biology, School of Molecular and Cellular Biology, Faculty of Biological Sciences, University of Leeds, Leeds, LS2 9JT, UK
[3]Astbury Centre for Structural Molecular Biology, School of Physics & Astronomy and Biomedical Sciences, Faculty of Engineering & Physical Sciences and Biological Sciences, University of Leeds, Leeds, LS2 9JT, UK

[#]These authors contributed equally

*Correspondence should be addressed to M.Foglizzo@leeds.ac.uk, E.Zeqiraj@leeds.ac.uk, marcus.wilson@ed.ac.uk

Supplementary Figure S1: Reconstitution of modified nucleosomes and quality control of assays.

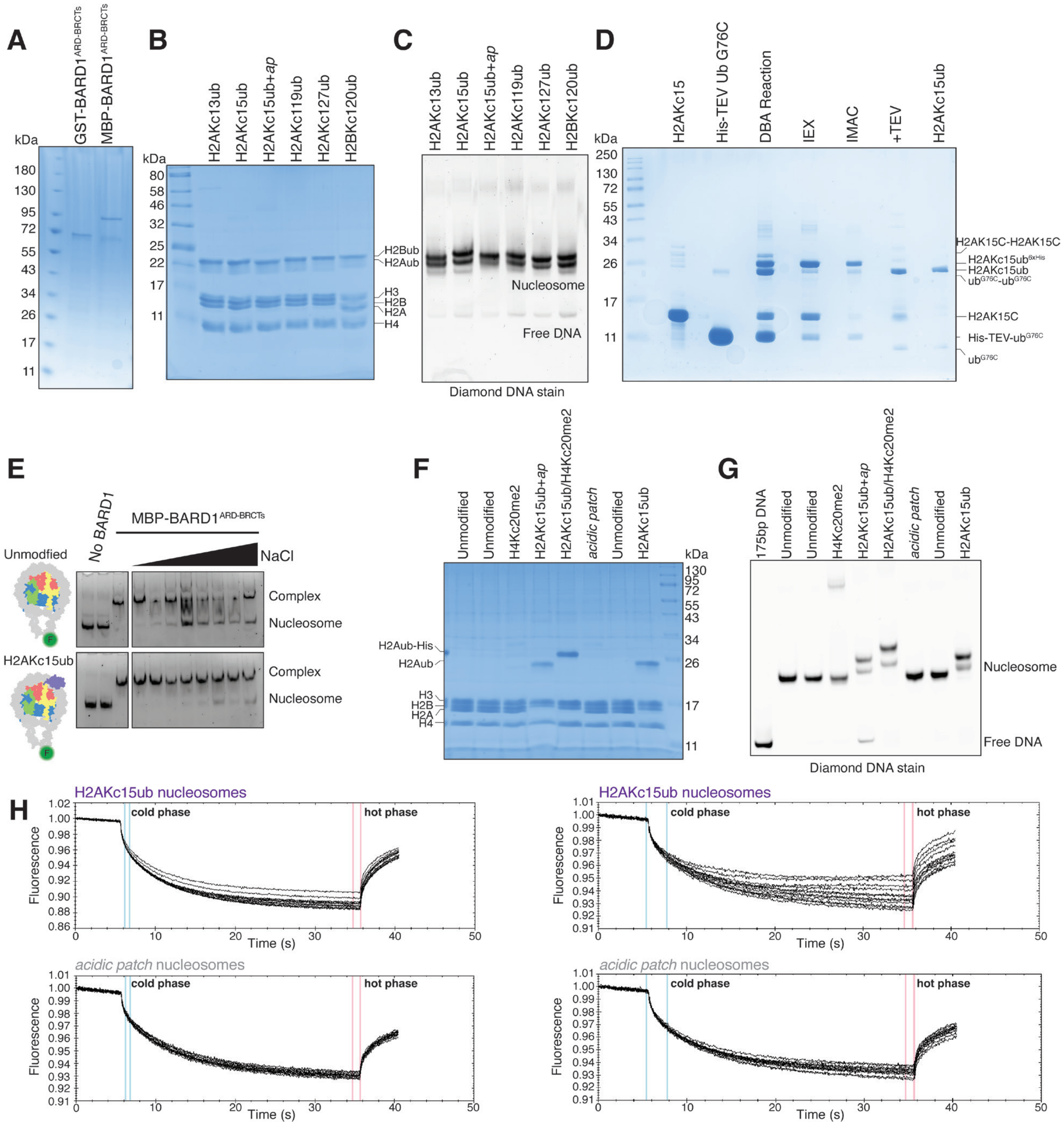

A. SDS-PAGE gel showing GST-BARD1$^{ARD-BRCTs}$ and 6xHis-MBP-BARD1$^{ARD-BRCTs}$ proteins used in pulldown, EMSA, ubiquitylation and MST assays. Gels were stained and imaged with colloidal Coomassie stain.

B. SDS-PAGE gel showing various ubiquitylated nucleosomes used in GST pulldown experiments. The gel was stained and imaged with colloidal Coomassie. Ub = ubiquitin, AP = acidic patch.

C. Native-PAGE gel showing various ubiquitylated nucleosomes used in GST pulldown experiments. The gel was stained and imaged with Diamond DNA stain; shift in mobility suggests DNA is bound to histones. Doublet commonly observed for ubiquitylated nucleosomes.

D. SDS-PAGE gel representing the different steps required for chemical ubiquitylation of H2A mutants. Cysteine mutants are introduced at the desired site of ubiquitylation (e.g. H2AK15C), and to the terminal glycine of 6xHis-TEV-tagged ubiquitin (ub^{G76C}). H2AK15C and 6xHis-ub^{G76C} are mixed with di-bromo acetone (DBA), incubated at 4°C for ~30 minutes, and quenched with β-mercaptoethanol before being purified by ion exchange (IEX) and immobilised metal affinity (IMAC) chromatography. TEV protease is used to remove the 6xHis tag from the ubiquitylated histone, and a second round of IMAC removes the protease and uncleaved samples. The gel was stained and imaged with colloidal Coomassie.

E. EMSA experiments assessing 6xHis-MBP-$BARD1^{ARD\text{-}BRCTs}$ protein binding to recombinant nucleosomes under increasing concentrations of NaCl. Ubiquitin specificity is increased at more physiological salt concentrations. Nucleosomes were wrapped with 5' FAM-labelled DNA and chemically ubiquitylated at H2A position 15. The complexes were pre-formed with 2.3 nM nucleosome variants and 640 nM BARD1 in 100 mM NaCl (seen to fully shift to complex band in both unmodified and H2AKc15ub nucleosomes). The salt concentration was increased by 12.5 mM NaCl along the series from 112.5-200 mM NaCl, showing loss of complex band for unmodified nucleosomes at salt concentrations. Complexes were resolved by native-PAGE and imaged for fluorescein.

F. SDS-PAGE gel showing various ubiquitylated nucleosomes used in pulldown, EMSA, ubiquitylation and MST assays. The gel was stained and imaged with colloidal Coomassie.

G. Native-PAGE gel showing various nucleosomes used in pulldown, EMSA, ubiquitylation and MST assays. The gel was imaged for fluorescein. Ubiquitylated species run as multiple bands. Higher mobility of H2AKc15ub/H4Kc20me2 due to a 6xHis tag on H2Aub.

H. Raw MST traces of 6xHis-MBP-$BARD1^{ARD\text{-}BRCTs}$ (*Left*) and human Flag-$BRCA1^{\Delta Exon11}$:6xHis-BARD1 (*Right*) titrated against 5' FAM-labelled H2AKc15ub and acidic patch mutated nucleosomes. Refer to Figures 1F and 2B for details.

Supplementary Figure S2: BRCA-BARD1 complexes and fusion proteins bind and ubiquitylate nucleosomes.

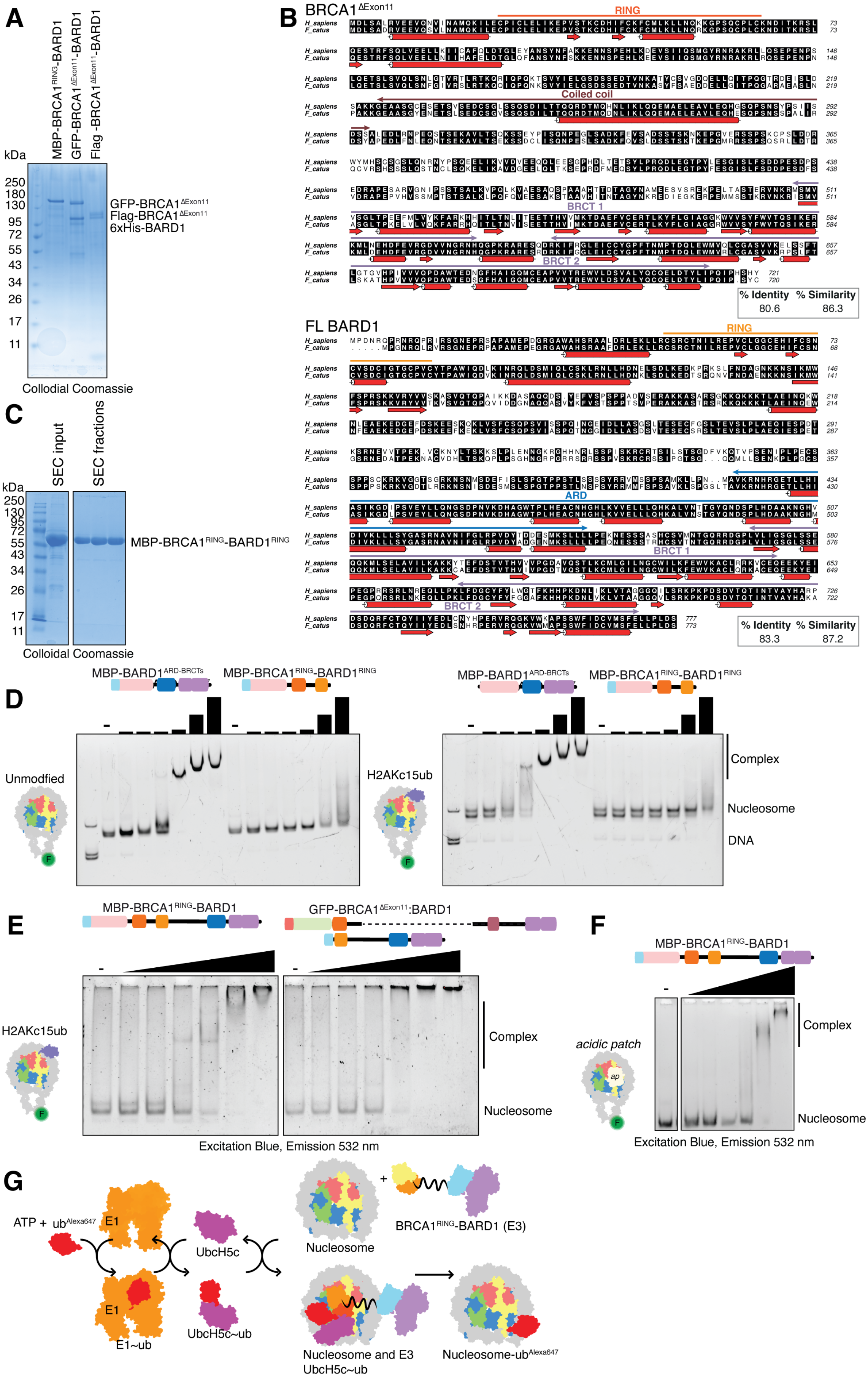

A. SDS-PAGE gel showing 6xHis-MBP-BRCA1^{RING}-BARD1,Flag-BRCA1$^{\Delta Exon11}$:6xHis-BARD1, and dStrepII-muGFP-BRCA1$^{\Delta Exon11}$:6xHis-BARD1 human proteins used in assays. The gel was stained and imaged with colloidal Coomassie stain.

B. Sequence alignments of BRCA1$^{\Delta Exon11}$ and FL BARD1 from *Homo sapie*ns and *Felis catus*. Identity between the proteins is indicated with black shading. Protein domains are labelled at the top of each alignment and colored as in Figure 2A; secondary structural elements are shown at the bottom. Tables reporting the percentage (%) of sequence identity and similarity between BRCA1$^{\Delta Exon11}$ and FL BARD1 species are also included. RING = Really Interesting New Gene, BRCT = BRCA1 C-terminus, ARD = Ankyrin Repeat Domain.

C. SDS-PAGE gel showing the SEC input and pool SEC fractions of the 6xHis-MBP-BRCA1^{RING}-BARD1^{RING} domains used in EMSA and ubiquitylation assays. The gels were stained and imaged with colloidal Coomassie.

D. Electrophoretic mobility shift assays (EMSAs) comparing interaction of 6xHis-MBP-BARD1$^{ARD\text{-}BRCTs}$ and 6xHis-MBP-BRCA1^{RING}-BARD1^{RING} with unmodified and H2AKc15ub nucleosome variants. Nucleosomes were wrapped with 5' FAM-labelled DNA (FAM = fluorescein), purified and limiting amounts (2.3 nM) were incubated with increasing concentrations (100-70,000 nM) of 6xHis-MBP-BARD1$^{ARD\text{-}BRCTs}$ and 6xHis-MBP-BRCA1^{RING}-BARD1^{RING} proteins. Lane marked with '–' indicates nucleosome alone. Complexes were resolved by native-PAGE and imaged using fluorescein filters. Concentration range is far larger than used elsewhere in this study in order to compare large differences between RING and ARD-BRCT affinities.

E. EMSA experiments using H2AKc15ub nucleosomes wrapped with 5' FAM-labelled DNA, and increasing concentrations (6.1-200 nM) of 6xHis-MBP-BRCA1^{RING}-BARD1 and human dStrepII-muGFP-BRCA1$^{\Delta Exon11}$:6xHis-BARD1. Complexes were resolved by native-PAGE and imaged for fluorescein.

F. EMSA experiments using H2AKc15ub+*ap* nucleosomes wrapped with 5' FAM-labelled DNA, and increasing concentrations (8-512 nM) of 6xHis-MBP-BRCA1^{RING}-BARD1. Complexes were resolved by native-PAGE and imaged for fluorescein.

G. Schematic of the ubiquitylation assays performed using recombinant nucleosomes and BRCA1^{RING}-BARD1. Nucleosomes were combined in assay buffer with ATP, ubiquitin (ub), ubiquitin^{Alexa647} (ub^{Alexa647}), UbcH5c (E2), and BRCA1^{RING}-BARD1 (E3). Reactions were initiated by addition of E1 enzyme.

Supplementary Figure S3: Generating K63-linked di-ubiquitin chains on H2AKc15ub.

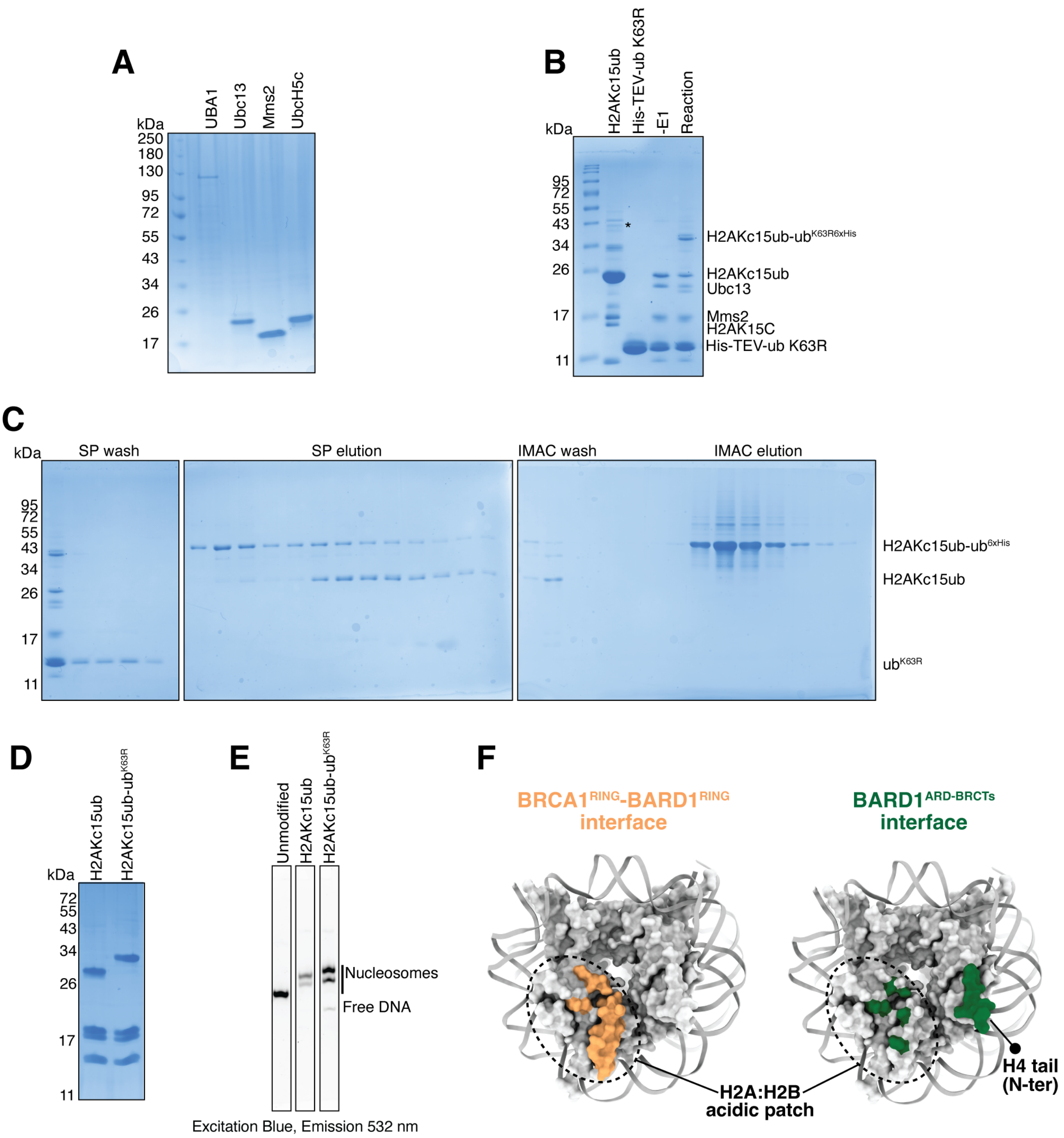

A. SDS-PAGE gel showing the E1 activating and E2 conjugating enzymes used in E3 ligase activity assays or for the enzymatic ubiquitylation of histone H2A. The gel was stained and imaged with colloidal Coomassie.

B. SDS-PAGE gel showing a summary of the E2-mediated Lys-63 ubiquitylation reaction on chemically modified H2AKc15ub. Chain elongation beyond di-ub was blocked using the K63R ubiquitin mutant. Additional bands indicated with asterisks correspond to by-products formed during the chemical ubiquitylation reaction, subsequently purified away in **C**. The gel was stained and imaged with colloidal Coomassie.

C. Purification process of H2AKc15ub-ub$^{K63R\text{-}6xHis}$. Ion exchange (IEX) chromatography was used to remove unreacted ubiquitin and to partially separate the H2AKc15ub and H2AKc15ub-ub$^{K63R\text{-}6xHis}$ species. H2AKc15ub-ub$^{K63R\text{-}6xHis}$ was further purified from H2AKc15ub by immobilised metal affinity chromatography (IMAC), taking advantage of a 6xHis tag fused to ub^{K63R}. The tag was removed using TEV protease prior to wrapping the histone into octamers. Gels were stained and imaged with colloidal Coomassie.

D. SDS-PAGE gel showing H2AKc15ub and H2AKc15ub-ub^{K63R} octamers. Gels were stained and imaged with colloidal Coomassie.

E. Native-PAGE gels showing unmodified, H2AKc15ub and H2AKc15ub-ub^{K63R} nucleosomes. Ubiquitylated nucleosomes run as doublets due to alternate conformations of covalently attached ubiquitin. Nucleosomes containing K63 chains are larger and have lower overall electrophoretic mobility. Gels were imaged for fluorescein.

F. Cartoon model of mono-nucleosome core particles (from this study) showing overlapping interfaces of the two BRCA1-BARD1 interaction modules. The H2A:H2B and H4 surfaces involved in BRCA1-BARD1 RINGs (*Left*) and BARD1 ARD-BRCTs (*Right*) interactions are coloured in light orange and green respectively. The histone octamer and nucleosomal DNA are coloured in light and dark grey.

Supplementary Figure S4: Cryo-EM structure determination and validation of Cat BRCA1$^{\Delta Exon11}$:BARD1 in complex with H2AKc15ub nucleosomes.

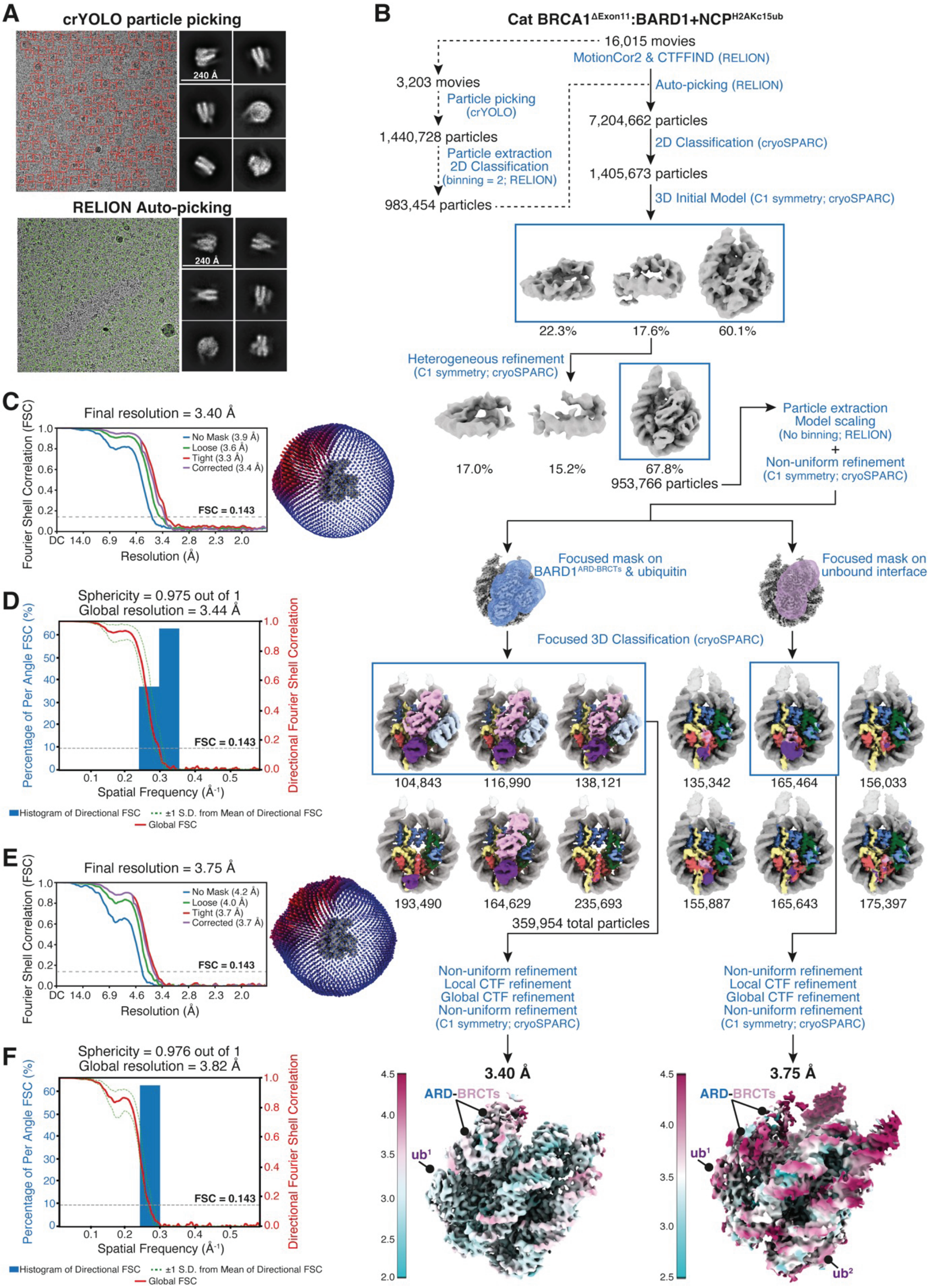

A. Representative micrographs (out of 16,015 movies collected) and 2D class averages (out of nine and 95 selected images following crYOLO and RELION particle picking, respectively) for the cryo-EM dataset of BRCA1$^{\Delta Exon11}$:BARD1 in complex with H2AKc15ub nucleosomes. Red boxes and green circles (240 pixels each) indicate picked particles.

B. Flow-chart of data processing strategy. Boxed 3D initial models and refined class were selected for subsequent processing. 3D classes obtained from Focused 3D Classification are coloured by corresponding chains (as in Figure 4B), and those used to obtain the final 3D reconstructions are highlighted with blue boxes. The final maps at global resolutions of 3.40 Å and 3.75 Å are shown and coloured by local resolution. Refer to Supplementary Table S3 for details.

C. FSC curves (*Left*) and Euler angle distribution (*Right*) in the final 3D reconstruction of the BARD1$^{ARD\text{-}BRCTs}$:NCP^{H2AKc15ub} complex obtained at 3.40 Å. The resolution was calculated using the gold-standard FSC cut-off at 0.143 frequency; rod heights are proportional to the number of particles in each direction.

D. Plot of the directional FSC (3DFSC), which represents a measure of directional resolution anisotropy, for the 3.40 Å BARD1$^{ARD\text{-}BRCTs}$:NCP^{H2AKc15ub} map. Global FSC (red lines) at a resolution of 3.44 Å, the spread of directional resolution values ± 1 standard deviation from the mean (area within the green dotted lines), and histograms of 100 directional resolutions evenly sampled over the 3DFSC (blue bars) are indicated. A sphericity of 0.975 was determined at the gold-standard FSC cut-off of 0.143 frequency, indicating no significant anisotropic angular distributions of particles.

E. FSC curves (*Left*) and Euler angle distribution (*Right*) in the final 3D reconstruction of the BARD1$^{ARD\text{-}BRCTs}$:NCP^{H2AKc15ub} complex obtained at 3.75 Å. The resolution was calculated as described in **C**; rod heights are proportional to the number of particles in each direction.

F. 3DFSC plot as described in **D**, but for the 3.75 Å BARD1$^{ARD\text{-}BRCTs}$:NCP^{H2AKc15ub} map. Global FSC (red lines) at a resolution of 3.82 Å, the spread of directional resolution values ± 1 standard deviation from the mean (area within the green dotted lines), and a histogram of 100 directional resolutions evenly sampled over the 3DFSC (blue bar) are indicated. A sphericity of 0.976 was determined at the gold-standard FSC cut-off of 0.143 frequency, indicating no significant anisotropic angular distributions of particles.

Supplementary Figure S5: Structural analyses of the BARD1$^{ARD-BRCTs}$:NCP^{H2AKc15ub} complex.

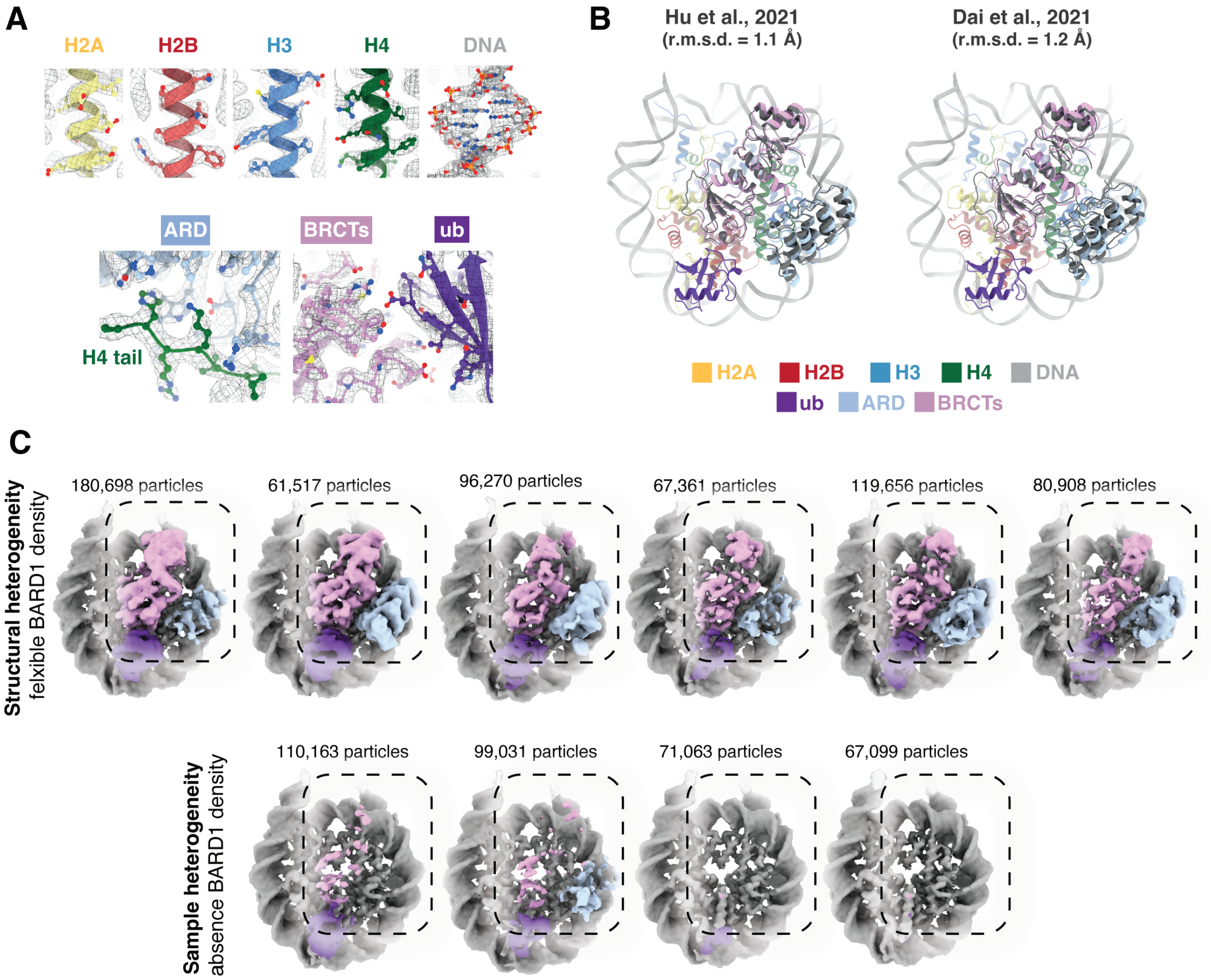

A. Representative regions of the BARD1$^{ARD-BRCTs}$:NCP^{H2AKc15ub} cryo-EM density map (see Figure 4B) for the different components of the complex. The densities for histones, DNA, BARD1 ARD, BARD1 BRCTs and ubiquitin are depicted at a contour level of 0.244, and the corresponding structural models coloured as in Figure 4B. ARD = Ankyrin Repeat Domain, BRCTs = BRCA1 C-terminus, ub = ubiquitin.

B. Overlays between the cryo-EM structures of BARD1$^{ARD-BRCTs}$:NCP^{H2AKc15ub}. The structure reported in this study (at 3.40 Å resolution) is coloured as in Figure 4B, while the corresponding complexes reported previously (PDB ID: 7LYC; PDB ID: 7E8I) are coloured grey. Structures are shown in cartoon models.

C. Selected focused 3D classification of the BARD1 ARD-BRCTs and ubiquitin regions reveals flexibility of the ARD and BRCT domains relative to the nucleosome particle. 3D classes and particle numbers are indicated; ubiquitin, and the BARD1 ARD and BRCT domains are

coloured as in Figure 4B. Dashed boxes highlight the region of interest of the BARD1 ARD-BRCTs between various 3D classes.

Supplementary Figure S6: Biochemical reagents generated to probe the clamp hypothesis of BRCA1-BARD1 interaction.

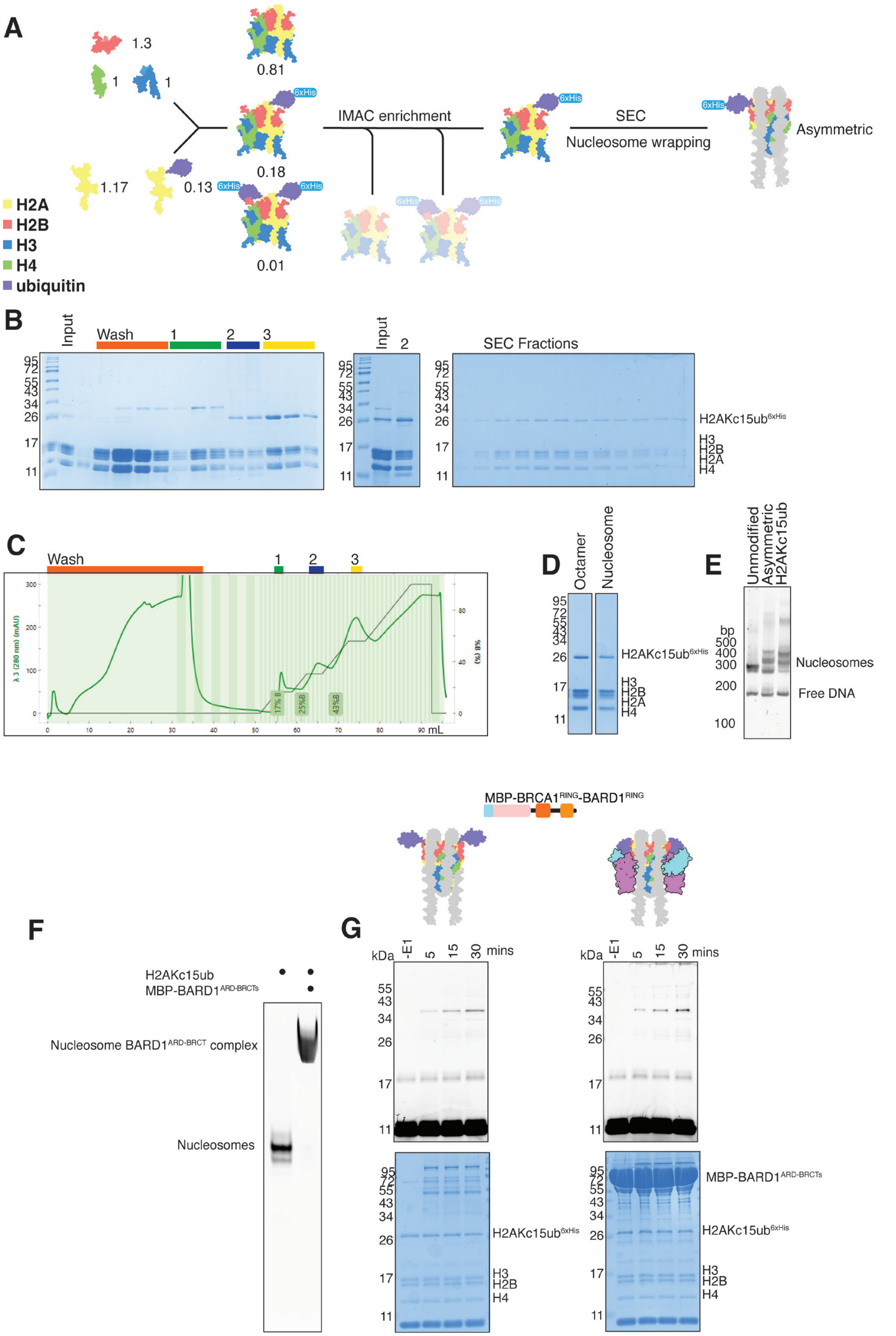

A. Schematic of the method used for making asymmetrically-modified ubiquitylated octamers. The concentration of 6xHis-tagged ubiquitylated histone was limited in refolding, leading to excess of unmodified octamer, which could be removed due to lack of a 6xHis tag. Numbers next to individual histones indicate molar ratios of individual histone components mixed before dialysis into refolding buffer. Numbers next to folded octamers indicate the expected ratio of each species after refolding and after IMAC respectively. Octamers were subjected to SEC after IMAC.

B. SDS-PAGE gels showing the purification process of asymmetric octamers. Input is the mixture prior to the IMAC step. Wash samples, and peaks 1, 2 and 3 are shown in the IMAC trace in **C**. Asterisk corresponds to contaminant band removed in purification.

C. IMAC trace of the gradient elution of asymmetric octamers using an elution buffer containing 300 mM imidazole. Peak fractions run on a gel in **B** are indicated. %B of each peak is indicated on the x-axis at point of elution. Peak 2 was used for wrapping and enzymology (Figure 4D).

D. SDS-PAGE of asymmetric octamers and nucleosomes used in EMSA and ubiquitylation experiments. The gel was stained and imaged with colloidal Coomassie.

E. Native-PAGE gel showing unmodified, asymmetric and H2AKc15ub nucleosomes used in EMSA and MST experiments. The gel was stained and imaged with DNA Diamond stain. Multiple bands observed for asymmetric nucleosomes are due to mixed species comprising mostly asymmetric modified, with minor unmodified and symmetrically modified nucleosomes.

F. Native-PAGE gel showing H2AKc15ub nucleosomes before ubiquitylation assay, after one hour incubation with saturating amounts (70 µM) of 6xHis-MBP-BARD1$^{ARD\text{-}BRCTs}$ protein.

G. Ubiquitylation assays assessing 6xHis-MBP-BRCA1^{RING}-BARD1^{RING} E3 ligase activity on H2AKc15ub nucleosomes that were (*Right panel*) or were not (*Left panel*) pre-incubated with 70 µM 6xHis-MBP-BARD1$^{ARD\text{-}BRCTs}$ protein. Samples were taken prior to addition of E1 (-E1) and at 5, 15, and 30 minute time points, and quenched by addition of 2x SDS loading buffer. Samples were resolved on SDS-PAGE gels and imaged for Alexa647 signal, before staining with Coomassie. Top panel shows the Alexa647 signal, and lower panel a colloidal Coomassie stain of the total protein.

Supplementary Figure S7: Generation of di-nucleosomes for assessing BRCA1-BARD1 binding.

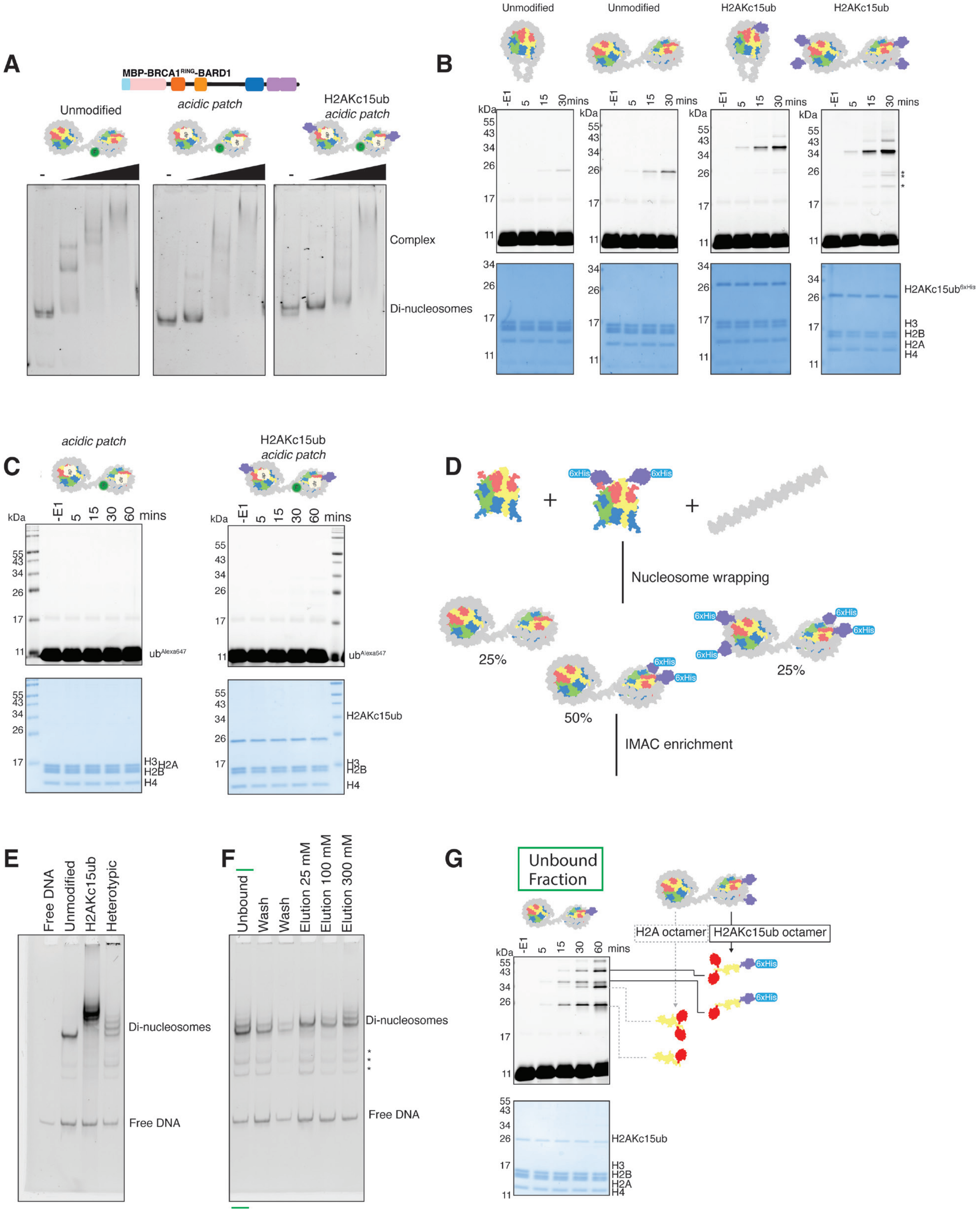

A. EMSA experiments using various di-nucleosomes to assess the role of the acidic patch in binding. Di-nucleosomes wrapped with 5' FAM-labelled DNA, and increasing concentrations

(10-100 nM) of 6xHis-MBP-BRCA1^{RING}-BARD1. Complexes were resolved by native-PAGE and imaged for fluorescein.

B. Ubiquitylation assays comparing 6xHis-MBP-BRCA1^{RING}-BARD1 E3 ligase activity on unmodified and H2AKc15ub mono- and d-nucleosomes. Unmodified and H2AKc15ub di-nucleosomes data are same as shown in Figure 5C. Samples were taken prior to addition of E1 (-E1) and at 5, 15, and 30 minute time points, and quenched by addition of 2x SDS loading buffer. Samples were resolved on SDS-PAGE gels and imaged for Alexa647 signal, before staining with Coomassie. Top panel shows the Alexa647 signal, and lower panel a colloidal Coomassie stain of the total protein. Asterisks indicate off target ubiquitylation activity.

C. Ubiquitylation assays assessing 6xHis-MBP-BRCA1^{RING}-BARD1 E3 ligase activity on acidic patch mutant (*Left*) and acidic patch mutant+H2AKc15ub (*Right*) di-nucleosomes. Samples were taken prior to addition of E1 (-E1) and at 5, 15, 30 and 60 minute time points, and quenched by addition of 2x SDS loading buffer. Samples were resolved on SDS-PAGE gels and imaged for Alexa647 signal, before staining with Coomassie. Top panel shows the Alexa647 signal, and lower panel a colloidal Coomassie stain of the total protein.

D. Model of heterotypic di-nucleosomes generated by mixing equimolar ratios of H2AKc15ub-6xHis octamer and unmodified octamer in di-nucleosome wrapping, prior to IMAC-based purification.

E. Native-PAGE gel showing the wrapping of unmodified, H2AKc15ub and unmodified+H2AKc15ub heterotypic di-nucleosomes. Multiple bands expected due to presence of doublet-forming ubiquitylated species with and without a 6xHis tag, which affects mobility on native-PAGE.

F. Native-PAGE illustrating the purification protocol used to enrich for heterotypic di-nucleosomes from **E**. Mixed population of fully ubiquitylated, partially ubiquitylated (only single octamer) and non-ubiquitylated di-nucleosome samples were incubated with Ni-NTA beads for two hours and then washed with IMAC A buffer, before being eluted from the beads with increasing concentrations of imidazole (*Right*). Recovered di-nucleosomes were buffer exchanged into nucleosome buffer and used in ubiquitylation assays.

G. Ubiquitylation assays testing 6xHis-MBP-BRCA1^{RING}-BARD1 ligase activity on the unbound fraction obtained from the heterotypic di-nucleosome purification shown in **F**, which was enriched for a nucleosome specie containing a single ubiquitin. Samples were taken prior to addition of E1 (-E1) and at 5, 15, 30 and 60 minute time points, and quenched by addition of 2x SDS loading buffer. Samples were resolved on an SDS-PAGE gel and imaged for Alexa647 signal, before staining with Coomassie. Top panel shows the Alexa647 signal, and lower panel a colloidal Coomassie stain of the total protein.

Supplementary Figure S8: Cryo-EM structure determination and validation of the isolated BRCA1$^{\Delta Exon11}$:BARD1 dataset.

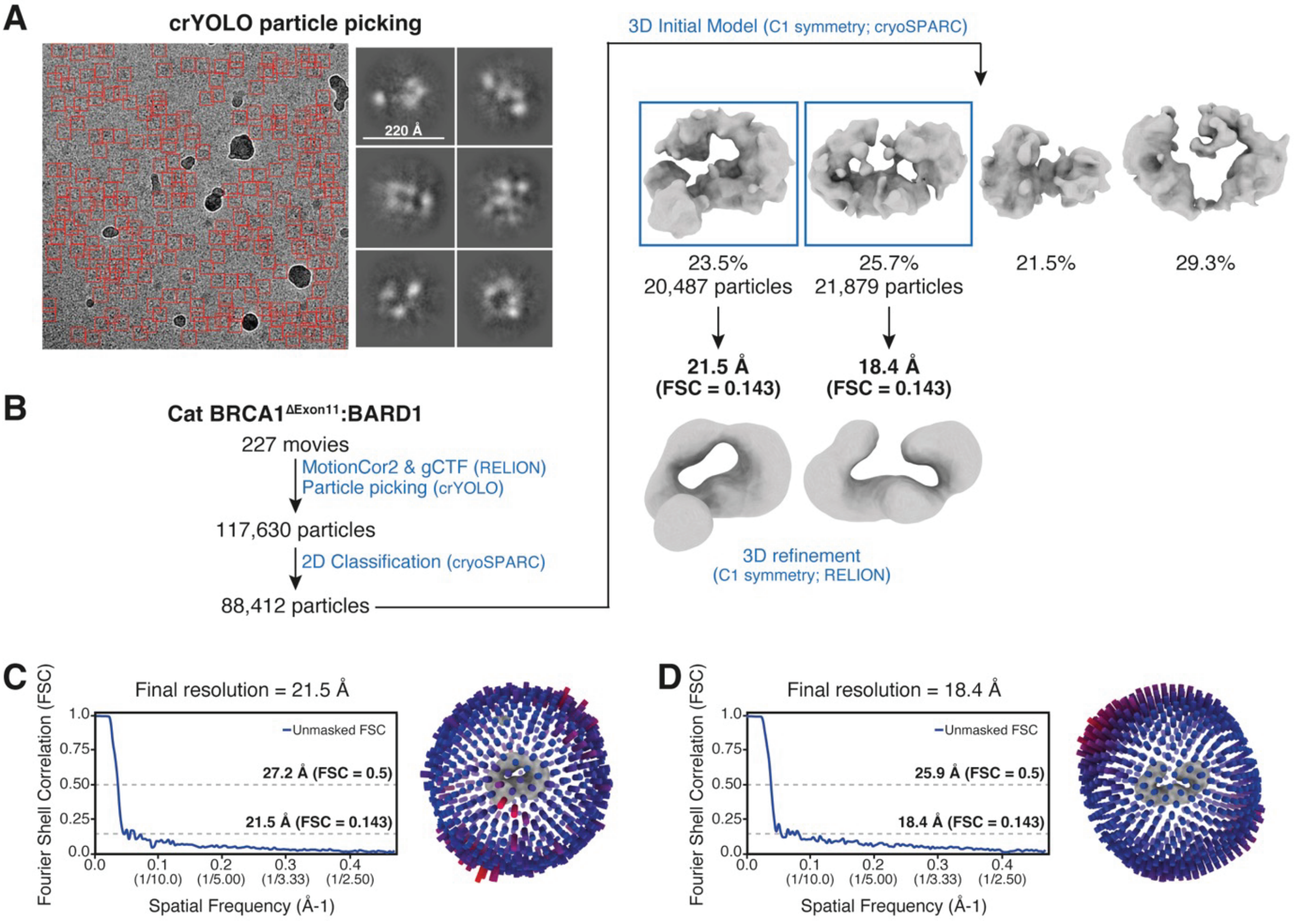

A. Representative micrographs (out of 227 movies collected) and 2D class averages (out of 91 selected images) for the isolated BRCA1$^{\Delta Exon11}$:BARD1 complex dataset. Red boxes (220 pixels) indicate picked particles.

B. Flow-chart of data processing strategy. Boxed 3D initial models were selected for subsequent processing. The final maps at the reported global resolutions of 21.5 Å (*Left*; "closed" state) and 18.4 Å (*Right*; "open" state) are shown. Refer to Supplementary Table S3 for details.

C. Unmasked FSC curve (*Left*) and Euler angle distribution (*Right*) in the final 3D reconstruction of the BRCA1$^{\Delta Exon11}$:BARD1 "closed" state. The resolution was calculated using the gold-standard FSC cut-off at 0.143 frequency; the reported resolution at the FSC cut-off frequency of 0.5 is also included. Rod heights are proportional to the number of particles in each direction.

D. As in **C**, but for the BRCA1$^{\Delta Exon11}$:BARD1 "open" state.

Supplementary Figure S9: Atomic force microscopy (AFM) imaging of di-nucleosomes in the absence and presence of the BRCA1$^{\Delta Exon11}$:BARD1 complex.

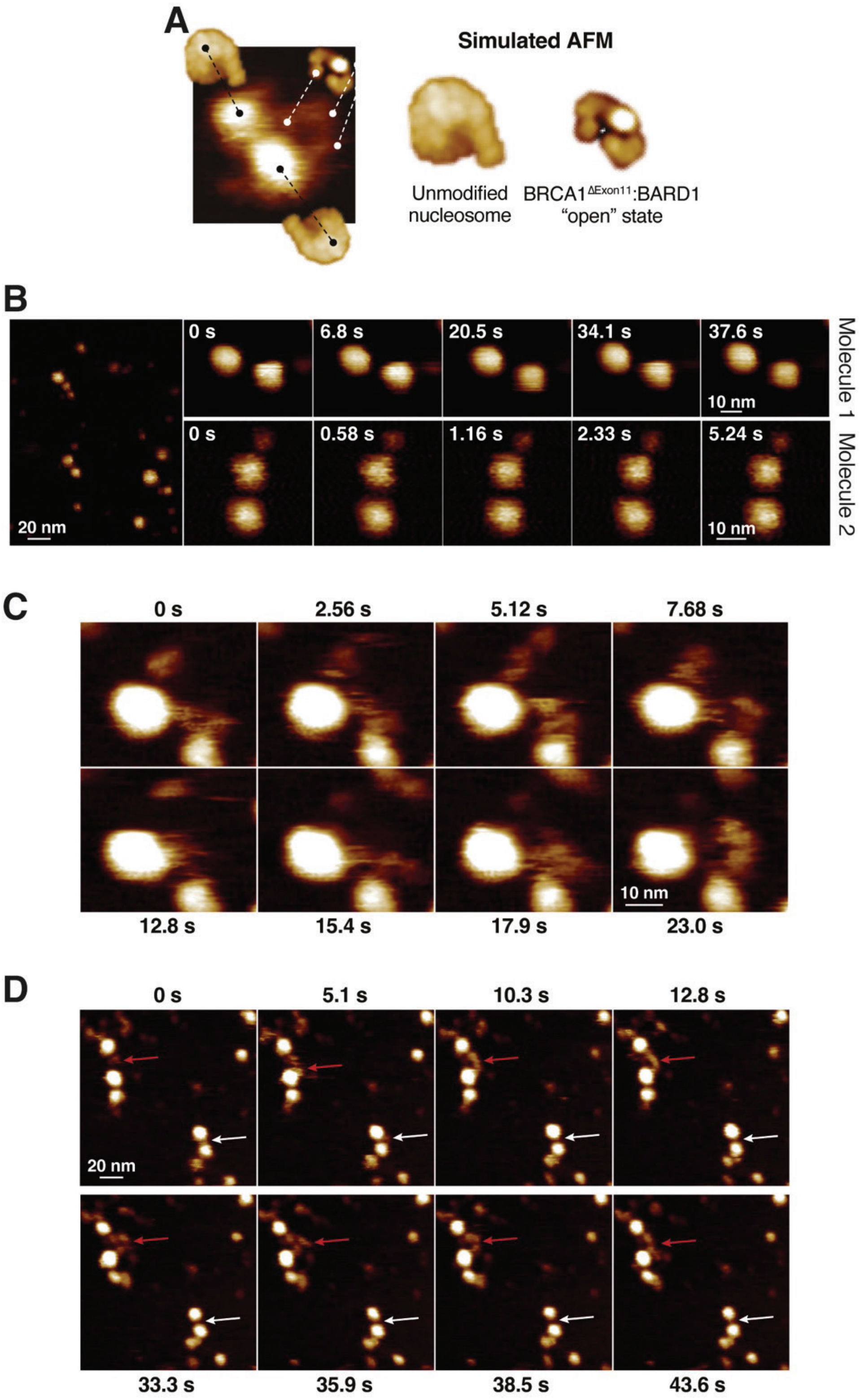

A. Atomic force microscopy (AFM) simulation of unmodified di-nucleosomes (PDB ID: 7PF4) and of the "open" state cat BRCA1$^{\Delta Exon11}$:BARD1 map (Figure 6A, Supplementary Figure

S8B). Tip radius and sampling values used in simulation AFM were 1 nm and 3 pixels/nm, respectively. Refer to Figure 6D for details.

B. High speed (HS)-AFM of di-nucleosome particles (*Left*) and close-up views captured over time (*Right*). Zoomed-in views were taken from a different area than the overview image.

C. HS-AFM of human BRCA1$^{\Delta Exon11}$:BARD1 in complex with unmodified di-nucleosomes imaged over time.

D. Overview images obtained by HS-AFM of human BRCA1$^{\Delta Exon11}$:BARD1 interactions with unmodified di-nucleosomes over time. Red arrows indicated BRCA1$^{\Delta Exon11}$:BARD1 bridging between a mono- and a di-nucleosome; white arrows indicate BRCA1$^{\Delta Exon11}$:BARD1 bridging across a di-nucleosome.

Supplementary Figure S10: Formation and testing of H4 methylated mono- and di-nucleosomes.

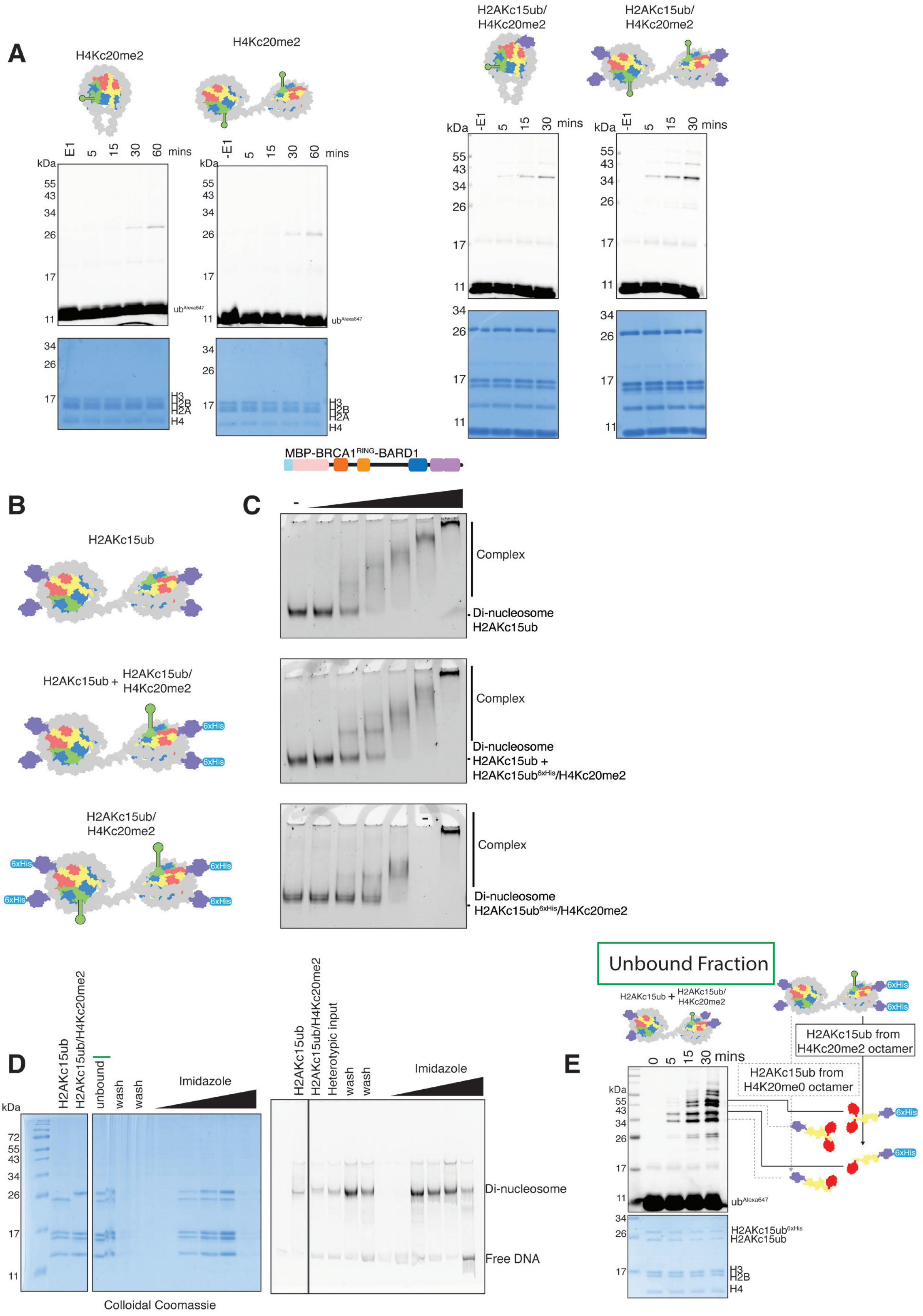

A. Ubiquitylation assays assessing 6xHis-MBP-BRCA1^{RING}-BARD1 E3 ligase activity on H4Kc20me2 and H2AKc15ub/H4Kc20me2 mono- and di-nucleosomes. Samples were taken prior to addition of E1 (-E1) and at 5, 15, 30 and 60 minute or at 5, 15, and 30 minute time points, and quenched by addition of 2x SDS loading buffer. Samples were resolved on SDS-PAGE and imaged for Alexa647 signal, before staining with Coomassie. Top panel shows the Alexa647 signal and lower panel a colloidal Coomassie stain of the total protein.

B. Schematic representation of different di-nucleosomes generated to assess the role of H4Kc20me2 in a di-nucleosome context.

C. EMSA experiments using various di-nucleosomes wrapped with 5' FAM-labelled DNA, and increasing concentrations (8-512 nM) of 6xHis-MBP-BRCA1^{RING}-BARD1. Complexes were resolved by native-PAGE and imaged for fluorescein. '–' indicates empty lane.

D. SDS- (*Left*) and native-PAGE (*Right*) gels showing the purification process of H2AKc15ub, H4Kc20me2 and H2AKc15ub/H4Kc20me2 heterotypic di-nucleosomes. Heterotypic nucleosomes were incubated with Ni-NTA beads for two hours, and then washed with IMAC A buffer, before being eluted from the beads with increasing concentrations of imidazole. Recovered di-nucleosomes were buffer exchanged into nucleosome buffer, then used in ubiquitylation assays.

E. Ubiquitylation assays testing 6xHis-MBP-BRCA1^{RING}-BARD1 activity on the unbound fraction obtained from the heterotypic di-nucleosome purification shown in **D**. Samples were taken prior to addition of E1 (-E1), and at 5, 15, and 30 minute time points, and quenched by addition of 2x SDS loading buffer. Samples were resolved on an SDS-PAGE gel and imaged for Alexa647 signal, before staining with Coomassie. Top panel shows the Alexa647 signal, and lower panel a Coomassie stain of the total protein.

Supplementary Table S1 – Summary of the BRCA1 and BARD1 expression constructs used in this study

Expression Construct	Species	Protein 1 (name)	Protein 1 (aa)	Tag	Protein 2 (name)	Protein 2 (aa)	Tag	Expression System
BARD1 ARD-BRCTs	Human	BARD1	425-777	N-ter. GST	-	-	-	*E. coli*
BARD1 ARD-BRCTs	Human	BARD1	425-777	N-ter. 6xHis-MBP	-	-	-	*E. coli*
BRCA1^{RING}-BARD1^{RING}	Human	BRCA1	1-100	N-ter. 6xHis-MBP	BARD1	26-140	none	*E. coli*
BRCA1^{RING}-BARD1	Human	BRCA1^{RING}	1-100	N-ter. 6xHis-MBP	BARD1	26-777	none	*E. coli*
BRCA1$^{\Delta11}$: BARD1	Human	BRCA1$^{\Delta11}$	1-223, 1366-1863	N-ter. Flag	BARD1	1-777	N-ter. 6xHis	*Tni*
BRCA1$^{\Delta11}$: BARD1	Human	BRCA1$^{\Delta11}$	1-223, 1366-1863	N-ter. dStrepII-muGFP	BARD1	1-777	N-ter. 6xHis	*Tni*
BRCA1$^{\Delta11}$: BARD1	Cat	BRCA1$^{\Delta11}$	1-223, 1369-1873	N-ter. Flag	BARD1	1-773	N-ter. 6xHis	*Tni*
BRCA1$^{\Delta11}$: BARD1	Cat	BRCA1$^{\Delta11}$	1-223, 1369-1873	N-ter. dStrepII-muGFP	BARD1	1-773	N-ter. 6xHis	*Tni*

Supplementary Table S2 – Summary of dissociation constant values (K_d) calculated by MST

	BARD1$^{ARD\text{-}BRCTs}$	BRCA1$^{\Delta Exon11:}$BARD1
**Nucleosome variants**		
Unmodified	0.218 µM ± 0.044 µM	0.093 µM ± 0.026 µM
H2AKc15ub	0.072 µM ± 0.010 µM	0.034 µM ± 0.005 µM
H2AKc15ub-ub	0.024 µM ± 0.004 µM	0.013 µM ± 0.002 µM
H4Kc20me2	0.964 µM ± 0.889 µM	0.365 µM ± 0.302 µM
Acidic Patch	ND	ND
H2AKc15ub + Acidic Patch	ND	ND
H2AKc15ub + H4Kc20me2	0.298 µM ± 0.157 µM	0.118 µM ± 0.055 µM

Supplementary Table S3 – Cryo-EM data collection, refinement and validation statistics

	**BARD1$^{ARD-BRCTs}$:NCP^{H2AKc15ub} (EMD-16859; EMD-17928 PDB ID: 8OFF)**		**BRCA1$^{\Delta Exon11}$:BARD1 (EMD-16869; EMD-16870)**	
**Data Collection**				
Microscope	TFS Titan KRIOS		TFS Titan KRIOS	
Detector	Falcon IV		Falcon III	
Voltage (keV)	300		300	
Mode	Counting		Integrating	
Pixel size (Å)	0.86		1.065	
Magnification (x)	96,000		75,000	
Electron dose ($e^-/Å^2$)	36.4		73	
Exposure (s)	5		1.7	
EPU Frames (Nr)	172		50	
Electron dose per frame ($e^-/Å^2$)	0.8		1.46	
EER Fractions	45		-	
No. of movies	16,015		227	
Defocus range (μm)	-1.7 to -3.1		-1.5 to -3.0	
**Data Processing**	**Map 1**	**Map 2**	**Map 1**	**Map2**
Symmetry Point Group	C1	C1	C1	C1
Initial particle number	7,204,662	7,204,662	117,630	117,630
Final particle number	359,954	165,464	20,487	21,879
Map resolution (Å)	3.40	3.75	21.5	18.4
FSC threshold	0.143	0.143	0.143	0.143
**Refinement**				
Model resolution (Å)	3.0			
FSC threshold	0.5			
Map sharpening *B* factor ($Å^2$)	179.2			
Model composition				
Chains	13			
Non-hydrogen atoms	14,428			
Protein residues	1,146			
Nucleotide	289			
Water	0			
Ligands	0			
B factors ($Å^2$)				
Protein (min/max/mean)	40.38/129.24/59.00			
Nucleotide (min/max/mean)	64.61/134.19/90.89			
Water	-			
Ligands	-			
Map:model CC				
CC (mask)	0.82			
CC (box)	0.83			
CC (peaks)	0.81			
CC (volume)	0.83			
R.m.s. deviations				
Bond lengths (Å)	0.007			
Bond angles (°)	0.624			
**Validation**				
MolProbity score	2.43			
Clashscore	5.34			
Rotamers outliers (%)	6.83			
Cß outliers (%)	0			
Peptide plane				
Cis proline/general (%)	0/0			
Twisted proline/general (%)	0/0			
CaBLAM outliers (%)	2.91			
Ramachandran plot				
Favored (%)	91.27			
Allowed (%)	8.73			
Outliers (%)	0			

Supplementary Movie Legends

Supplementary Movie S1

Overlaid 3D class montage of BARD1 ARD-BRCTs on H2AKc15ub nucleosome particle. 3D classes are depicted in Supplementary Figure S5C.

Supplementary Movie S2

High speed-atomic force microscopy (HS-AFM) imaging of unmodified di-nucleosomes in the presence of the human BRCA1$^{\Delta Exon11}$:BARD1 complex. Still frames are shown in Figure 6D.
